# Protection against influenza-induced Acute Lung Injury (ALI) by enhanced induction of M2a macrophages: possible role of PPARγ/RXR ligands in IL-4-induced M2a macrophage differentiation

**DOI:** 10.3389/fimmu.2022.968336

**Published:** 2022-08-16

**Authors:** Archana Gopalakrishnan, John Joseph, Kari Ann Shirey, Achsah D. Keegan, Marina S. Boukhvalova, Stefanie N. Vogel, Jorge C. G. Blanco

**Affiliations:** ^1^ Department of Microbiology and Immunology, University of Maryland, School of Medicine, Baltimore, MD, United States; ^2^ Sigmovir Biosystems, Inc., Rockville, MD, United States; ^3^ Center for Vascular and Inflammatory Diseases, University of Maryland, School of Medicine, Baltimore, MD, United States

**Keywords:** influenza, M1 macrophage, M2 macrophage, PPARG, Acute Lung Injury

## Abstract

Many respiratory viruses cause lung damage that may evolve into acute lung injury (ALI), a cytokine storm, acute respiratory distress syndrome, and ultimately, death. Peroxisome proliferator activated receptor gamma (PPARγ), a member of the nuclear hormone receptor (NHR) family of transcription factors, regulates transcription by forming heterodimers with another NHR family member, Retinoid X Receptor (RXR). Each component of the heterodimer binds specific ligands that modify transcriptional capacity of the entire heterodimer by recruiting different co-activators/co-repressors. However, the role of PPARγ/RXR ligands in the context of influenza infection is not well understood. PPARγ is associated with macrophage differentiation to an anti-inflammatory M2 state. We show that mice lacking the IL-4Rα receptor, required for M2a macrophage differentiation, are more susceptible to mouse-adapted influenza (A/PR/8/34; “PR8”)-induced lethality. Mice lacking *Ptgs2*, that encodes COX-2, a key proinflammatory M1 macrophage mediator, are more resistant. Blocking the receptor for COX-2-induced Prostaglandin E_2_ (PGE_2_) was also protective. Treatment with pioglitazone (PGZ), a PPARγ ligand, increased survival from PR8 infection, decreased M1 macrophage gene expression, and increased PPARγ mRNA in lungs. Conversely, conditional knockout mice expressing PPARγ-deficient macrophages were significantly more sensitive to PR8-induced lethality. These findings were extended in cotton rats: PGZ blunted lung inflammation and M1 cytokine gene expression after challenge with non-adapted human influenza. To study mechanisms by which PPARγ/RXR transcription factors induce canonical M2a genes, WT mouse macrophages were treated with IL-4 in the absence or presence of rosiglitazone (RGZ; PPARγ ligand), LG100754 (LG; RXR ligand), or both. IL-4 dose-dependently induced M2a genes *Arg1*, *Mrc1, Chil3, and Retnla*. Treatment of macrophages with IL-4 and RGZ and/or LG differentially affected induction of *Arg1* and *Mrc1 vs*. *Chil3* and *Retnla* gene expression. In PPARγ-deficient macrophages, IL-4 alone failed to induce *Arg1* and *Mrc1* gene expression; however, concurrent treatment with LG or RGZ + LG enhanced IL-4-induced *Arg1* and *Mrc1* expression, but to a lower level than in WT macrophages, findings confirmed in the murine alveolar macrophage cell line, MH-S. These findings support a model in which PPARγ/RXR heterodimers control IL-4-induced M2a differentiation, and suggest that PPARγ/RXR agonists should be considered as important tools for clinical intervention against influenza-induced ALI.

## Graphical Abstract

Upper panel: *In vivo* model depicting regulation of lung-induced pathology by M1 and M2 macrophages. In absence or in the presence of low levels of influenza-induced IL-4, treatment with PPARγ agonist, Pioglitazone (PGZ), enhances M2 macrophage polarization and reduces influenza-induced lung disease. Lower panel: *In vitro* IL-4-dependent regulation of canonical M2a macrophages genes is selectively modulated by ligands specific for PPARγ/RXR heterodimers. PPARγ/RXR heterodimers gain access to a peroxisome proliferator activated receptor response element (PPRE) in the promoter of canonical M2a macrophage genes, inducing their expression in a ligand-independent manner. However, in the presence of ligands for both PPARγ and RXR [Rosiglitazone (RGZ) and LG100764 (LG), respectively], liganded heterodimers selectively enhance the expression of some M2a genes (*Arg1 and Mrc1*) without affecting the others (*Retnla and Chil3*).

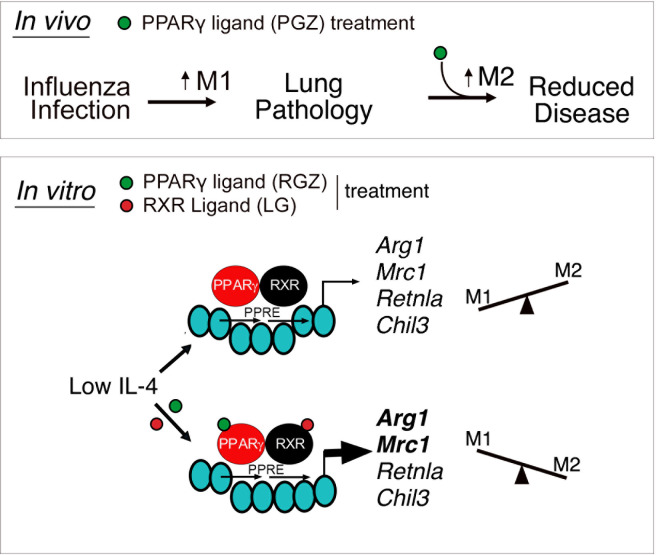

## Introduction

Macrophage differentiation is driven by metabolic changes that alter cellular functionality. At one extreme, LPS and IFN-γ drive glycolysis, with greatly reduced oxidative phosphorylation, leading to “M1” macrophages that are highly microbicidal and produce proinflammatory mediators (rev. in 1). While facilitating pathogen clearance, an overexuberant M1 macrophage response may cause tissue damage. In contrast, “M2a” macrophages differentiate in response to exogenous or endogenous IL-4 or IL-13 (via the shared IL-4Rα), are strongly dependent on oxidative phosphorylation for energy, and produce proteins, including anti-inflammatory cytokines, that mediate wound healing (rev. in 1–7). However, the M1/M2 paradigm is more nuanced than originally thought (8–12): macrophage subpopulations exhibit different thresholds for activating these metabolic programs, *i.e.*, lung alveolar and interstitial macrophages acquire distinct metabolic capacities in response to infection (13–16), and subpopulations of M2 macrophages (*e.g.*, M2a, M2b, M2c) have been identified based on distinct inducers and transcriptional profiles (rev. in 12). It has been proposed that the distinct transcriptional programs induced during macrophage polarization to an M2 phenotype are controlled by epigenomic modifications at specific gene promoters that enhance transcription (17, 18)

In response to Respiratory Syncytial Virus (RSV) infection, both alveolar macrophages and thioglycollate-elicited macrophages elicit an early (by ~8 h) proinflammatory M1 response, followed by production of endogenous IL-4 and IL-13 that, in turn, drive a strong M2a response (~48 h peak) that counters RSV-induced M1 macrophages and ALI (6). Induction of M2 macrophages by IL-4 or RSV coincided with upregulation of Peroxisome Proliferating Activated Receptor gamma (PPARγ) (6), a transcription factor that heterodimerizes with Retinoid X Receptor (RXR) and is required for differentiation of macrophages towards an M2 polarization phenotype (18–22)*. Pparg* mRNA was not induced in RSV-infected TLR4^-/-^ mice and macrophages, and RSV-infected TLR4^-/-^ mice exhibited increased peribronchiolar and perivascular inflammation compared to WT mice (6). This suggests that M2 macrophage polarization is the host’s response to the damaging effects of RSV-induced M1 macrophages. This is supported by our findings that therapeutic administration of agents that induce M2 macrophages (*e.g*., rosiglitazone (RGZ), a PPARγ agonist, IL-4/anti-IL-4 complexes, and azithromycin) enhanced resolution of RSV-induced ALI in mice (23). Mice lacking alveolar macrophages (24) or PPARγ in myeloid cells (25) exhibited decreased survival in response to influenza infection, suggesting that PPARγ in alveolar macrophages may protect against influenza-induced disease. The studies presented herein provide clear evidence for a role for M2 macrophages in the resolution of influenza-induced disease and provide new insights into the regulation of M2 macrophage gene expression by engaging PPARγ and RXR receptor heterodimers with the ligands for one, the other or both concurrently as a therapeutic strategy.

## Materials and methods

### Reagents

A stock solution of pioglitazone hydrochloride (PGZ Actos, Takeda Pharmaceutical of America) was prepared in pyrogen-free saine (for mouse studies) or in DMSO (cotton rat studies). The EP4 (prostaglandin E_2_ (PGE_2_) receptor) antagonist, AP1531, was kindly provided by Dr. Steven Orndorff (Ariel Pharmaceuticals, Broomfield, CO) and was made up in pyrogen-free saline. Rosiglitazone (RGZ) purchased from Cayman Chemical Company (Ann Arbor, MI) and LG100754 (LG) from Sigma Aldrich (St. Louis, MO) were reconstituted to 100 μM in DMSO (final concentration of 0.001% DMSO). TSPO agonist, FGIN-1-27 was purchased from Tocris Bioscience (Minneapolis, MN) and reconstituted to 50 mM in DMSO (final concentration of 0.02%). Recombinant mouse IL-4 (*E. coli*-derived) was purchased from R&D Systems (Minneapolis, MN) and reconstituted to a stock solution of 50 μg/ml in PBS. All reagents used for *in vitro* experiments were stored at -20° C.

### Animals

Six to 8 week old, male and female WT C57BL/6J and BALB/cByJ mice, and COX2^-/-^ mice were purchased from the Jackson Laboratory (Bar Harbor, ME). IL-4Rα^-/-^ mice (BALB/c background; originally provided to Dr. Achsah Keegan by Nancy Noben-Trauth) (Rockville, MD) and William Paul (Bethesda, MD) were bred homozygously at the University of Maryland, Baltimore (UMB). Homozygous floxed PPARγ mice crossed into a transgenic mouse containing the *Cre* gene under control of the M lysozyme promoter (PPARγ flox^+/+^/Cre^+/+^) to delete the *Pparg* gene in lysozyme-producing cells (*e.g*., macrophages, neutrophils) (20) (referred to as “PPARγ^cKO^ mice;” C57BL/6 background) were kindly provided to Dr. Stefanie Vogel by Dr. Mary Jane Thomassen (East Carolina University) and were bred homozygously at the University of Maryland, Baltimore. Four-six week old, male and female inbred cotton rats (*Sigmodon hispidus*) were bred at Sigmovir Biosystems, Inc. All animal experiments were conducted with institutional IACUC approvals.

### Viruses

Mouse-adapted H1N1 influenza A/PR/8/34 virus (“PR8”) (ATCC, Manassas, VA) was grown in the allantoic fluid of 10-day old embryonated chicken eggs as described (26) and was kindly provided by Dr. Donna Farber (Columbia University). The seed of the non-adapted influenza A/California/07/2009(H1N1) (A/California) was obtained from the CDC and grown in eggs in house. The stocks of virus contained 2 X 10^8^ TCID_50_/ml.

### *In vivo* experiments in mice and cotton rats

For survival experiments, some mice (WT C57BL/6J and COX2^-/-^) mice were infected with an LD_90_ of mouse-adapted influenza virus PR8 (~7500 TCID_50_ i.n., 25 µl/nare) (27, 28). In other experiments where we anticipated increased sensitivity to infection, WT C57BL/6J and PPARγ^cKO^ mice were infected with an LD_40_ (~3000 TCID_50_ i.n., 25 µl/nare) of PR8, while WT BALB/cByJ and IL-4Rα^-/-^ were infected with an LD_10_ (~1500 TCID_50_). All infected mice were monitored for survival for 14 days post-infection. For *in vivo* treatments, mice were infected with the indicated PR8 dose on day 0 and treated with vehicle (saline), AP1531 (180 μg/mouse intravenously (i.v.), or PGZ (6.3 mg/kg/mouse intraperitoneally (i.p.) daily from days 2-6 p.i. and survival was followed for 14 days. Survival data were analyzed using a Mantel-Cox log-rank test. For harvest assays, mice were infected with PR8 on day 0 and treated with vehicle (saline) or PGZ daily from days 2-6 p.i. Three hours after the last treatment, mice were euthanized and their lungs extracted for mRNA gene expression by qRT-PCR (see below). No difference in sensitivity to PR8 was observed between C57BL/6 and BALB/cByJ mice.

Cotton rats were challenged at the initiation of the experiment (day 0) with 1 x 10^6^ TCID_50_ of A/California pH1N1 virus, intranasally. On day 1 post-infection, animals were treated with 3 mg/kg of PGZ (1:10 DMSO in PBS) or vehicle control (1:10 DMSO:PBS) i.p., with treatments continued daily until day 5, when the animals were sacrificed. Lungs were dissected *en bloc*, with the left lobes used for RNA extraction and qRT-PCR gene expression analysis, and the right lobes inflated and fixed with 10% formalin, paraffin-embedded and 4 µm sections stained with Hematoxylin and Eosin for histopathology analysis. Slides were scored blindly for 4 different lung pathology parameters: peribronchiolitis (inflammatory cells surrounding a bronchiole), perivasculitis (inflammatory cells surrounding a small blood vessel), alveolitis (inflammatory cells within alveolar spaces), and interstitial pneumonia (increased thickness of alveolar walls associated with inflammatory cells).

### Macrophage cell culture

Thioglycolate-elicited peritoneal macrophages were obtained and cultured as previously described (29–31). The murine alveolar macrophage cell line (MH-S) was purchased from ATCC (Manassas, VA) and cultured in RPMI 1640 media supplemented with 10% Fetal Bovine Serum, 2 mM L-glutamine, 1% Penicillin-Streptomycin, and 0.05 mM 2-mercaptoethanol.

### Quantitative real-time PCR

Total RNA isolation from lungs or macrophage cell culture and qRT-PCR were performed as previously described (6, 11, 27, 31, 32). Levels of mRNA for specific genes were first normalized to the level of the housekeeping gene encoding hypoxanthine phosphoribosyltransferase, *Hprt* (for mice), or β-actin, *Actb* (for cotton rats), in the same samples. Lung mRNA data is graphically presented as “fold-increase” over the relative gene expression measured in mock-infected lungs *in vivo* (2^−ΔΔCT^) (33). Murine gene expression was analyzed using Prism GraphPad Software by a one- or two-way ANOVA, as indicated, with Sidak’s multiple comparison *post-hoc* test, as indicated in the figure legends. Statistical analysis of data with only 2 treatment groups was performed by unpaired Student’s *t*-test.

*In vitro*, qRT-PCR was performed on total RNA from peritoneal macrophages and MH-S cells using transcript-specific primers as previously described (6). Data were analyzed using the non-transformed −ΔCT values after normalization to the housekeeping gene *Hprt*, and are graphically presented both as −ΔCT (left y-axis) and as “fold increase” (right y-axis) relative to the response of untreated RNA preparations from WT macrophages (2^−ΔΔCT^) (33). Statistical significance between treatment groups was determined using one- or two-way ANOVA, with differences between treatment groups evaluated by *post-hoc* comparisons (*e.g*., Tukey or Sidak *post-hoc* tests) using Prism GraphPad Software. A p value <0.05 was considered statistically significant.

## Results

### IL-4Rα^-/-^ mice are more susceptible than WT mice to influenza infection

**Figure 1:**
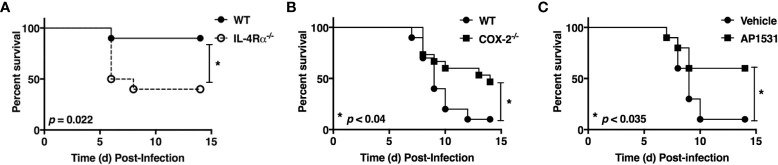
Opposing action of M2 vs. M1 macrophage activation on influenza (PR8)-induced lethality. **(A)** WT BALB/cByJ and IL-4Rα^-/-^ mice were infected i.n. on day 0 with ~1500 TCID_50_ A/PR/8/34 (PR8) (LD_10_) and survival was followed for 14 days. N = 10 mice/strain from 2 experiments. **(B)** WT C57BL/6J and COX2^-/-^ mice were infected i.n. on day 0 with ~7500 TCID_50_ PR8 (LD_90_) and survival was followed for 14 days. N = 15 mice/strain from 3 experiments. **(C)** WT C57BL/6J mice were infected with ~7500 TCID_50_ PR8 (LD_90_) on day 0 and then treated i.v. with vehicle or AP1531 (180 mg/mouse) daily from days 2-6 p.i. and survival was followed for 14 days. N = 10 mice/treatment from 2 separate experiments. Survival data was analyzed by the Mantel-Cox log rank test.

Influenza elicits a much more robust and prolonged M1 macrophage response *in vivo* than RSV, resulting in more severe ALI (27). In fact, it is very hard to measure M2 macrophage gene expression in lungs of influenza-infected mice unless they are sublethally-infected (34). **Figure 1A** shows that IL-4Rα^-/-^ mice (on a BALB/c background) that lack the IL-4Rα chain that is required for M2a macrophage differentiation induced by either IL-4 or IL-13 (35–37) are significantly more susceptible to an infectious dose of PR8 that is sublethal in wild-type (WT) mice.

### COX2, an M1 macrophage gene product, contributes to influenza-induced lethality

Cyclooxygenase-2 (COX2) is a key M1 macrophage proinflammatory mediator and administration of COX2-specific inhibitors mitigate ALI induced by RSV in mice and cotton rats (6, 38). Previous studies have shown that COX2^-/-^ mice are more resistant to influenza infection than WT mice or COX1^-/-^ mice (39); although, celecoxib, a COX2-specific inhibitor, failed to protect influenza-infected mice (40).

To revisit these conflicting findings, WT (C57BL/6J) or COX2^-/-^ mice were infected with PR8 (**Figure 1B**) or PR8-infected WT mice were treated therapeutically with saline or AP1531 (**Figure 1C**), an inhibitor of EP4, the receptor for COX2-derived prostaglandin E_2_ (PGE_2_). Both COX2^-/-^ mice and WT mice treated with AP1531 were significantly protected from PR8-induced lethality. Thus, this data strongly suggest that induction of M2 macrophages by IL-4/13 and/or blockade of the M1-associated gene, *Ptgs2* (or its downstream mediators), also dampens influenza-induced lung pathology and its associated lethality.

### Therapeutic administration of the PPARγ agonist, pioglitazone, protects mice from lethal influenza infection and decreases M1 and PPARγ gene expression

Therapeutic treatment of RSV-infected mice with rosiglitazone (RGZ), a PPARγ agonist, mitigated RSV-induced lung pathology (23). Pioglitazone (PGZ), another PPARγ agonist that is structurally related to RGZ (41), was administered to mice challenged with PR8 and to cotton rats challenged with a non-adapted human strain of influenza to determine if providing the ligand for PPARγ could be protective against the strong inflammatory response elicited by influenza infection.

**Figure 2:**
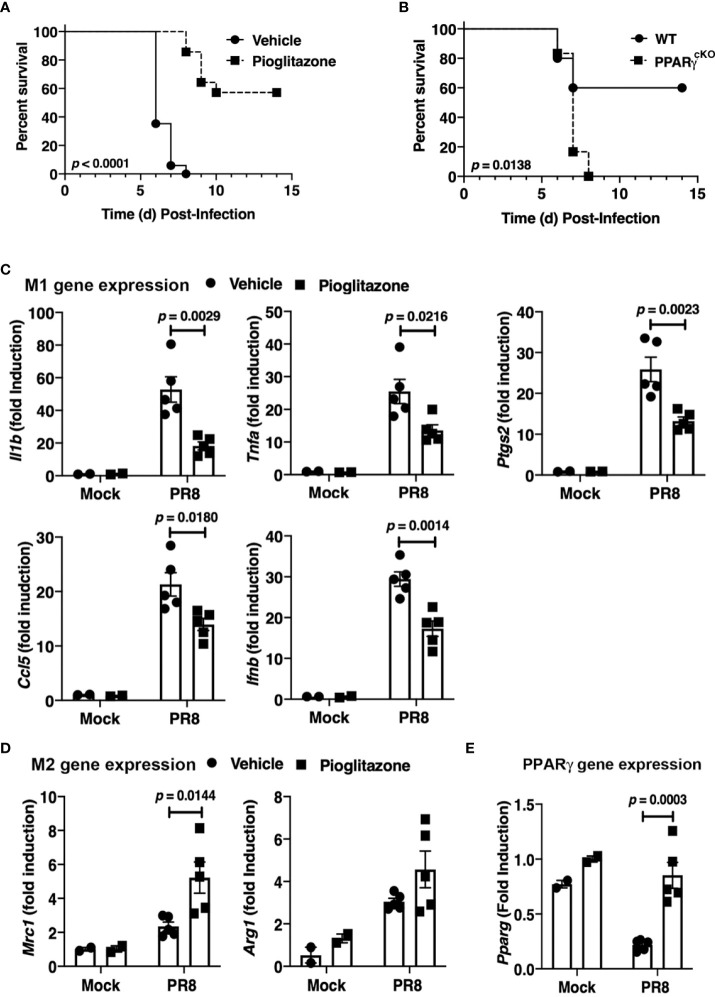
Protective role of PPARg in PR8-induced lethality. **(A)** WT C57BL/6J mice were infected with PR8 on Day 0 and treated on days 2-6 with vehicle or pioglitazone i.p. (6.3 mg/kg). Survival was monitored for 14 days. N = 7-8 mice per treatment group/experiment and 2 separate experiments. **(B)** WT C57BL/6J and PPARγ^cKO^ mice were infected with an LD_40_ of PR8 (~3000 TCID_50_; i.n.). Mice were monitored daily for survival for 14 days post-infection. N = 5-6 mice per strain/experiment and 2 separate experiments. Survival data was analyzed by the Mantel-Cox log rank test. **(C)** M1 and **(D)** M2 gene expression in lungs of mice infected with PR8 and mock-treated or treated with pioglitazone as in **(A)**. Lungs were harvested at day 6 post-infection. **(E)** PPARγ mRNA was decreased in response to PR8 infection, but treatment with pioglitazone resulted in normal PPARγ mRNA levels. Gene expression data was analyzed by a 2-way ANOVA with Sidak’s multiple comparison post-test.

WT C57BL/6J mice were infected with an LD_90_ of PR8, and then inoculated with PGZ (6.3 mg/kg) therapeutically, once daily for 5 consecutive days (*e.g.*, days 2 through 6 post-infection). PGZ-treated mice were significantly protected from lethality *(* **Figure 2A**; *p* < 0.0001).

To confirm these findings, homozygous floxed PPARγ mice were crossed into a transgenic mouse containing the *Cre* gene under control of the M lysozyme promoter (PPARγ flox^+/+^/Cre^+/+^) to delete the *Pparg* gene in lysozyme-producing cells (*e.g*., macrophages, neutrophils) (20). WT C57BL/6J and the myeloid PPARγ-deficient conditional knockout mice (“PPARγ^cKO^”) were compared for sensitivity to PR8 infection. **Figure 2B** illustrates that a dose of PR8 that killed 40% of WT mice resulted in lethality in 100% of myeloid-deficient PPARγ mice. Thus, myeloid cell PPARγ contributes to resistance against PR8 infection.

Therapeutic administration of PGZ to mice also resulted in the down-regulation of M1 macrophage gene expression (**Figure 2C**) and increased M2a macrophage gene expression of *Mrc1* in the lungs of mice (**Figure 2D**) on Day 6 post-infection, although the increased trend in the level of *Arg1* mRNA did not achieve statistical significance. Consistent with previous findings (25, 42), PR8 infection resulted in a decrease in *Pparg* mRNA expression in WT mice, while we further observed that treatment of PR8-infected mice with PGZ returned the level of *Pparg* mRNA to that of uninfected mice (**Figure 2E** **).**

### Therapeutic administration of the PPARγ agonist, pioglitazone, blunts proinflammatory gene expression, restores *Pparg* mRNA, and protects cotton rats from ALI in response to infection with non-adapted human influenza

**Figure 3:**
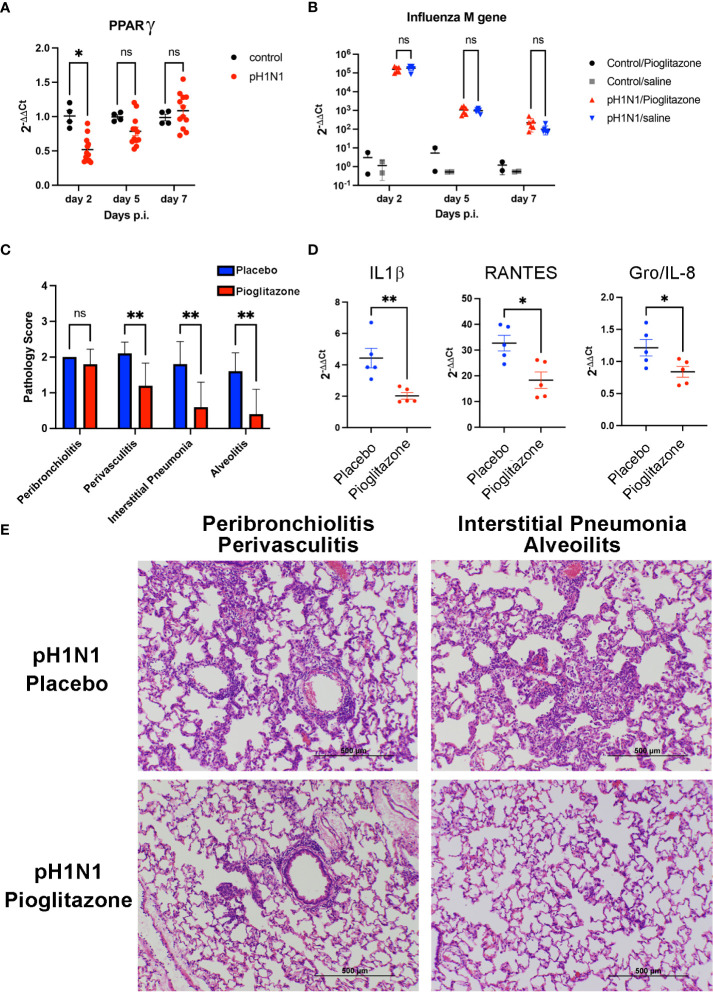
Pioglitazone treatment reduces pH1N1-induced lung pathology and inflammation without altering viral replication in cotton rats. **(A)** Expression of PPARγ mRNA in cotton rat lungs after pH1N1 (A/California/04/2009) influenza infection. *, p<0.05 by ANOVA (n = 4-10 cotton rats/time point). ns, not significant. **(B–E)**. Cotton rats infected with pH1N1 were treated daily starting 24 h after challenge with vehicle (Placebo) or 3 mg/kg of pioglitazone, i.p. until the day prior to sacrifice (day 4 or day 6 p.i). **(B)** Treatment with pioglitazone does not affect the kinetics of viral replication as measured by influenza M gene expression. ns, not significant. **(C–E)**. Cotton rats were euthanized on day 5 to determine lung pathology and expression of mRNA encoding inflammatory cytokines. **(C)** Pathology scores for the indicated features. **, p<0.01 by ANOVA followed by Sidak’s multiple comparisons test (n = 10 cotton rats/treatment group). ns, not significant. **(D)** qRT-PCR measurements for expression of IL-1β, RANTES, and Gro/IL-8 mRNA in lungs of infected and treated animals, as indicated. * and **, p<0.05 and p<0.01, respectively (unpaired *t*-test). **(E)** H&E-stained slides of cotton rat lungs showing representative features of pathology scored in C, *i.e.*, pioglitazone treatment induced a significant reduction in perivasculitis, interstitial pneumonia, and alveolitis, whereas no significant reduction in peribronchiolitis was observed.

Cotton rats (*Sigmodon hispidus*) represent a unique rodent species that is susceptible to many human viral respiratory pathogens, without the need for adaptation (43), including human strains of influenza (44). Young *S. hispidus* cotton rats (~6 wk) were infected with human influenza (1 x 10^6^ TCID_50_ A/California/04/2009, pH1N1) intranasally. In agreement with the results observed in PR8-infected mice (**Figure 2E**) and those previously reported (25, 42), influenza infection transiently inhibited expression of *Pparg* mRNA (**Figure 3A**) in the pH1N1-infected cotton rats. On day one post-infection, cotton rats were treated once daily with PGZ (3 mg/kg) for 5 days. PGZ treatment did not alter the kinetics of pH1N1 replication (**Figure 3B**). However, infected and PGZ-treated animals euthanized on day 5 post-infection, the peak of virus-induced pathology, showed greatly reduced lung inflammation (**Figure 3C**). Cotton rats infected with influenza and treated with PGZ also showed a significant reduction in the expression of proinflammatory M1 genes including IL-1β, RANTES, and Gro/IL-8 mRNA (**Figure 3D**), which was accompanied by a dramatic reduction of perivasculitis, interstitial pneumonia, and alveolitis (**Figures 3C, E**), and overall amelioration of the consolidated influenza-induced pneumonia.

### PPAR ligand and RXR ligand enhance a subset of IL-4-induced M2a genes *in vitro*

**Figure 4:**
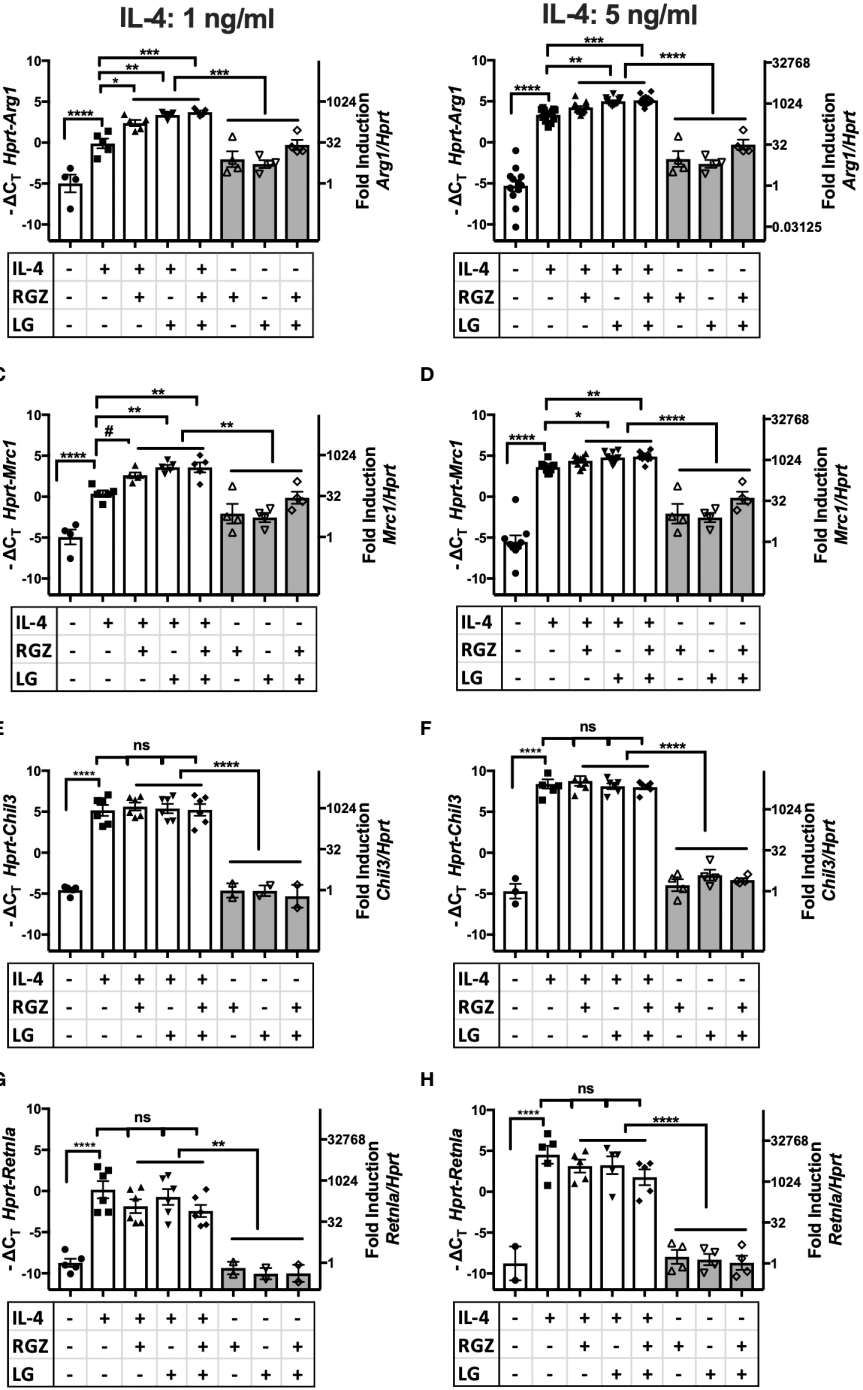
Effect of IL-4, PPARγ and RXR agonists on M2 macrophage gene expression using C57BL/6J peritoneal macrophages. Thioglycollate-elicited peritoneal macrophages were harvested from 6-8 week old WT C57BL/6J mice and stimulated with IL-4 at different concentrations **(A, C, E, G)** 1 ng/ml; **(B, D, F, H)** 5 ng/ml) in the absence or presence of 1 µM of rosiglitazone (RGZ) (PPARγ ligand) and/or 1 µM of LG100754 (LG) (RXR ligand) for 48hr, and RNA was processed as described in *Methods*. Gene expression was quamtified by qRT-PCR.Data were pooled from 5-9 independent experiments and is presented as -ΔC_T_ (left y-axis) and fold induction (2^-ΔΔCT^ value; right y-axis), mean ± SEM. Statistical analysis (one-way ANOVA with Tukey’s multiple comparison *post-hoc* test) was performed on the -ΔC_T_ values. ****p <0.0001, ***p<0.001, **p<0.01, *p<0.05, and ^#^p=0.054. ns, not significant.

PPARγ is a transcription factor required for induction of the M2a macrophage phenotype by IL-4 or IL-13 (20, 22), (rev. in 45). PPARγ forms heterodimers with Retinoid X Receptor (RXR), and their ligands are involved in the regulation of PPARγ/RXR heterodimer transcriptional function in the M2 gene expression program (46). We examined the effect of a PPARγ ligand and/or a RXR ligand on IL-4-mediated M2a macrophage polarization. Stimulation of thioglycollate-elicited murine peritoneal macrophages with recombinant murine IL-4 (ranging from 1 ng/ml to 20 ng/ml) upregulated canonical M2a gene expression, *i.e.*, *Arg1*, *Mrc1, Chil3, and Retnla* mRNA, as measured by qRT-PCR (**Figures 4A–H**, **Supplementary Figure 1**). At 1 ng/ml and 5 ng/ml IL-4, the presence of the PPARγ ligand, rosiglitazone (RGZ) and/or the RXR ligand, LG100754 (LG), enhanced expression of *Arg1* and *Mrc1* genes, although the levels of gene expression in the presence of RGZ were consistently less than when cells were stimulated with IL-4 in the presence of LG (*e.g*., IL-4/LG or IL-4/RGZ/LG) (**Figures 4A–D** **)**. When 20 ng/ml IL-4 was used to stimulate the macrophages, RGZ ± LG failed to enhance *Arg1* and *Mrc1* gene expression (**Supplementary Figure 1**). In the absence of IL-4, only the combined treatment with RGZ and LG increased *Arg1* and *Mrc1* gene expression significantly above levels induced by medium only (p<0.001), yet were significantly less than observed in the presence of IL-4.

In contrast to *Arg1* and *Mrc1*, neither IL-4-induced *Chil3* nor *Retnla* gene expression was further enhanced by RGZ and/or LG (**Figures 4E–H** **)**. Similar results were found when macrophages were pretreated with IL-4 for 24 h, followed by treatment with the PPARγ/RXR agonists for an additional 24 or 48 hr (data not shown).

**Figure 5:**
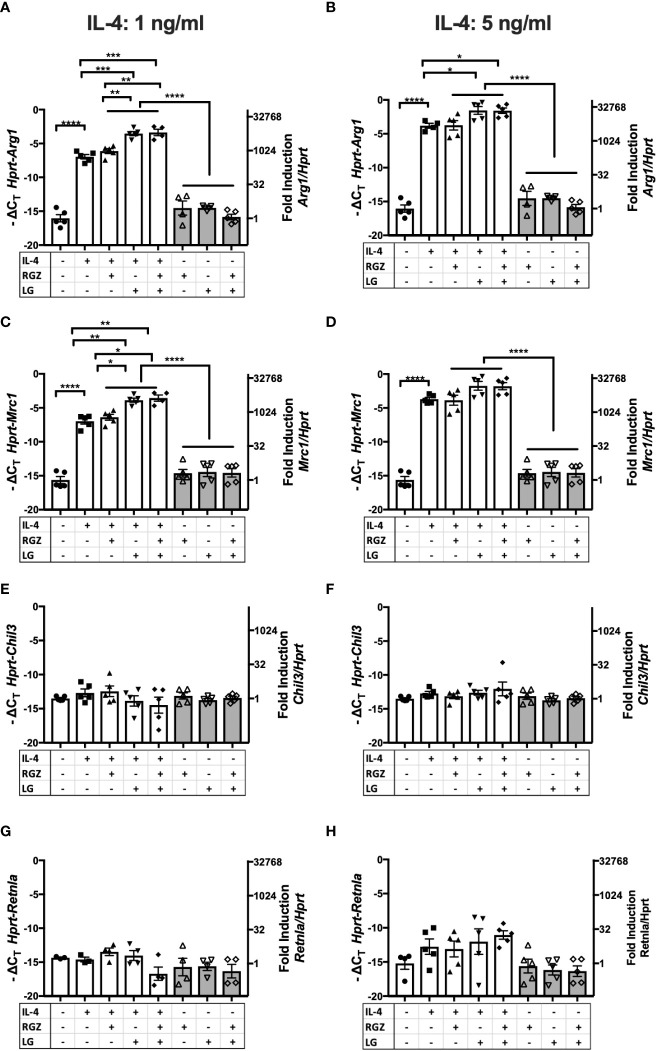
Effect of IL-4, PPARγ and RXR agonists on M2 macrophage gene expression using macrophages cell line MH-S. Murine alveolar macrophage cell line, MH-S was stimulated with IL-4 at different concentrations **(A, C, E, G)** 1 ng/ml; **(B, D, F, H)** 5 ng/ml) in the absence or presence of 1 µM of rosiglitazone (RGZ) (PPARγ ligand) and/or 1µM of LG100754 (LG) (RXR ligand) for 48 hr, and RNA was processed. Gene expression was quantified by qRT-PCR. Data were pooled from 3 independent experiments and is presented as -ΔC_T_ (left y-axis) and fold induction (2^-ΔΔ^CT value; right y-axis), mean ± SEM. Statistical analysis (one-way ANOVA with Tukey’s multiple comparison *post-hoc* test) was performed on the -ΔC_T_ values. ****p <0.0001, ***p<0.001, **p<0.01, and *p<0.05.

The ability of RGZ and LG to modulate IL-4-induced *Arg1* and *Mrc1* mRNA was confirmed using a murine alveolar macrophage cell line, MH-S (**Figures 5A–D** **)**, while IL-4 poorly induced *Chil 3* and *Retnla* mRNA in the MH-S cells and neither RG and/or LG increased their mRNA expression (**Figures 5E–H**).

### RXR ligand enhances IL-4-induced M2a gene expression in PPARγ-deficient macrophages

**Figure 6:**
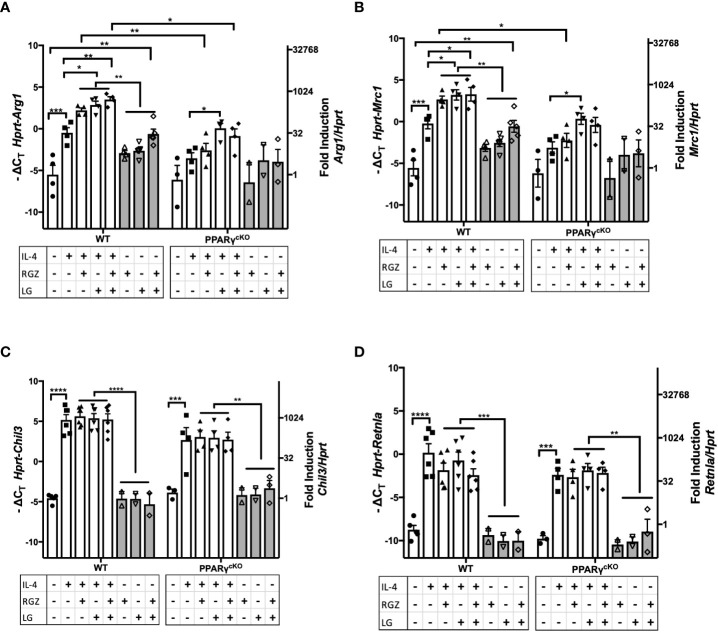
Effect of IL-4, PPARγ and RXR agonists on M2 macrophage gene expression in WT and PPARγcKO macrophages. Thioglycollate-elicited peritoneal macrophages were harvested from age-matched WT (C57BL/6J) and PPARγ ^cKO^ mice and stimulated with IL-4 at 1ng/ml in theabsence or presence of 1 μM of RGZ and/or 1 μM of LG for 48 hr, and RNA was processed. Gene expression of *Arg1* **(A)**, *Mrc1* **(B)**, *Chil3* **(C)**, and *Retnla* **(D)** were quantified by qRT-PCR. Data were pooled from 3 independent experiments and is presented as -ΔC_T_ (left y-axis) and fold induction (2^-ΔΔCT^ value; right y-axis), mean ± SEM. Statistical analysis (2-way ANOVA with Sidak’s multiple comparison *post-hoc* test) was performed on the -ΔC_T_ values. ****p <0.0001, ***p<0.001, **p<0.01, and *p<0.05.

PPARγ flox^+/+^/Cre^+/+^ (“PPARγ^cKO^”) mice do not express the *Pparg* gene in lysozyme-producing cells (20). We next compared expression of the M2a genes in WT *vs*. PPARγ^cKO^ macrophages. Peritoneal macrophages from age-matched WT and PPARγ^cKO^ mice were treated with IL-4 (1 ng/ml) in the absence or presence of RGZ and/or LG for 48 hr. As observed in **Figures 4**, **5**, and **Figures 6A, B** shows that IL-4 induced expression of *Arg1* and *Mrc1* in WT macrophages and this was enhanced by the presence of RGZ and/or LG. PPARγ^cKO^ macrophages exhibit a level of induction of *Arg1* and *Mrc1* gene expression in response to IL-4 that was not statistically significant. However, stimulation of PPARγ^cKO^ macrophages with LG significantly elevated IL-4-induced *Arg1* and *Mrc1* gene expression, but to a much lower extent than in the WT macrophages. This data indicates that even in the absence of PPARγ in macrophages, the RXR ligand, LG, can enhance IL-4-induced transcription of M2a genes.

Induction of *Chil3* and *Retnla* gene expression in macrophages by IL-4 was PPARγ-independent, as evidenced by WT levels of mRNA induced in PPARγ^cKO^ macrophages (**Figures 6C, D** **)** and the levels of *Chil3* and *Retnla* mRNA were not further augmented by the concurrent presence of RGZ and/or LG.

### The Translocator Protein agonist, FGIN-1-27, dampens IL-4-induced M2a polarization in peritoneal macrophages and in the MH-S alveolar macrophage cell line

**Figure 7:**
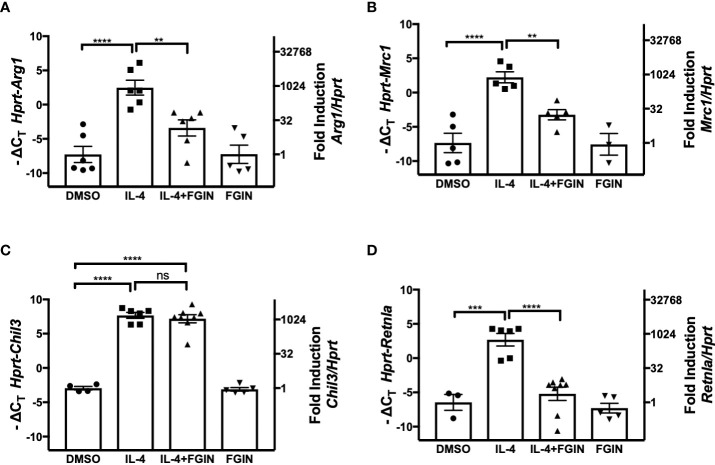
Effect of TSPO agonist on IL-4-induced M2 macrophage gene expression. Thioglycollate-elicited peritoneal macrophages were harvested from 6-8 week old C57BL/6J mice and stimulated with IL-4 at 5 ng/ml in the absence or presence of 10 µM of FGIN-1-27 (TSPO agonist) for 48 hr, and RNA was processed. Gene expression of *Arg1* **(A)**, *Mrc1* **(B)**, *Chil3* **(C)**, and *Retnla* **(D)** were quantified by qRT-PCR. Data from 3 independent experiments is presented as -ΔC_T_ (left y-axis) and fold induction (2^-ΔΔCT^ value; right y-axis), mean ± SEM. Statistical analysis (one-way ANOVA with Sidak’s multiple comparison *post-hoc* test) was performed on the -ΔC_T_ values. ****p <0.0001 and **p<0.01 ns, not significant.

TSPO is an 18-kDa mitochondrial outer membrane protein of microglia, the resident macrophages of the brain. In response to brain injury, microglia, like macrophages, first differentiate to a proinflammatory M1 phenotype, followed by differentiation to the anti-inflammatory M2 phenotype (rev. in 47, 48). During M2 polarization of primary microglial cell cultures by IL-4 treatment, expression of TSPO decreased, while the level of PPARγ was enhanced at the levels of both mRNA and protein (48). This study also showed that IL-4-induced expression of PPARγ, as well as the M2a markers encoded by *Arg1*, *Mrc1*, *Chil3*, and *Retnla*, were attenuated in microglial cells treated with the TSPO agonist, FGIN-1-27 (48). We sought to determine if the effects observed on M2 gene expression in microglial cells were also seen in macrophages. Peritoneal macrophages from WT mice were stimulated with IL-4 (5 ng/ml) in the absence or presence of TSPO agonist, FGIN-1-27 (10 μM), for 48 hr (**Figures 7A–D**). IL-4-induced upregulation of *Arg1*, *Mrc1*, and *Retnla* mRNA in peritoneal macrophages were significantly decreased in the presence of FGIN-1-27 (**Figures 7A, B, D**); however, FGIN-1-27 failed to down-regulate expression of IL-4-induced *Chil3* mRNA (**Figure 7C**). In the absence of IL-4, FGIN-1-27 alone did not modulate M2a gene expression. FGIN-1-27 also inhibited IL-4-induced *Arg1* and *Mrc1* in the MH-S cell line (**Supplemental Figures 2A, B** **).** Consistent with published findings on microglia, this data shows that TSPO agonist regulates a subset of IL-4-induced M2a gene expression in peritoneal macrophages.

## Discussion

Macrophages respond to environmental cues to exhibit a spectrum of responses tailored to the inciting stimulus (rev. in 3–7). On one extreme, LPS and IFN-γ elicit a shift of macrophage metabolism toward the glycolytic pathway, with oxidative phosphorylation being essentially shut off entirely (the “Warburg” effect), resulting in M1 macrophages that are highly microbicidal and produce proinflammatory mediators, *e.g.*, nitric oxide (NO), ROS, and proinflammatory cytokines (rev. in 1). However, an overexuberant M1 response can also result in significant tissue damage. In contrast to M1 macrophage differentiation, M2a macrophages develop in response to exogenous or endogenous IL-4 or IL-13, that share a common receptor chain, IL-4Rα (35), and mediate tissue repair and responses to helminths. In contrast to M1 macrophages, M2 macrophages depend on oxidative phosphorylation for energy with minimal changes to glycolysis over unstimulated macrophages, and produce high amounts of anti-inflammatory cytokines, but few proinflammatory mediators (rev. in 1–7). Recent studies have shown that distinct macrophage subpopulations exhibit different thresholds for activating these metabolic programs, *i.e.*, lung alveolar and interstitial macrophages acquire distinct metabolic capacities in response to infection (13–16). In fact, macrophage polarization has been proposed to take place in stages and is controlled by distinct transcriptional programs involving chromatin opening at specific M2 promoters, followed by signals that result in enhanced transcription (17, 18)

Our earlier studies focused on the roles of M1/M2 macrophages in RSV infection. Briefly, in response to RSV infection, lung macrophages, peritoneal macrophages, and macrophage cell lines all initiate an early and robust M1 proinflammatory response that peaks at ~8 h, followed by production of endogenous IL-4 and IL-13 that, in turn, drives a strong M2 response peaking at ~48 h post-infection and coincides with expression of PPARγ, a transcription factor that heterodimerizes with RXR and is required for induction of M2 macrophages (19–22). *In vivo*, we showed that development of the M2 response was IL-4Rα-, IFN-β-, and TLR4-dependent (6), and concluded that the transition from M1 to M2 macrophages during RSV infection reflects the host’s natural response that is intended to repair or counter the damage to the inflamed lung. Treatment of mice with three different agents reported previously to induce M2 differentiation mitigated RSV-induced lung pathology, showing that interventions that facilitate skewing of the host response to an M2 macrophage phenotype may be an effective strategy to counter M1 macrophage-induced inflammation and tissue damage (23).

Influenza infection results in a much stronger inflammatory lung response than RSV infection, and in fact, it is very difficult to detect M2 markers in mice unless they are infected with a sublethal dose of influenza (34). Gopal et al. (42) initially reported that infection of mice with a sublethal dose of influenza and concurrent treatment of mice with the PPARγ agonist, RGZ, from days 0-6 post-infection, decreased viral titers, neutrophil and monocyte infiltration into the bronchoalveolar lavage fluid, and proinflammatory cytokine production. Huang et al. (25) reported that in mice that lack PPARγ in lysozyme-expressing neutrophils and monocytes, a dose of PR8 that permitted 100% survival in WT control mice enhanced proinflammatory cytokine production and resulted in only ~60% survival in the PPARγ-deficient mice. However, they failed to observe an effect of myeloid PPARγ deficiency on induction of M2 genes including *Arg1* and *Retnla*. Our *in vivo* data extend these findings considerably: (1) the fact that IL-4Rα^-/-^ mice are more susceptible than WT mice to PR8 infection indicates that IL-4- and/or IL-13-induced signaling through their shared receptor to initiate M2a macrophage differentiation (rev. in 35) is required for relative resistance of WT mice, and (2) conversely, that COX2^-/-^ mice or mice treated therapeutically with a PGE_2_ receptor antagonist, are more resistant, indicates that this potent proinflammatory M1 gene contributes to influenza-induced disease. (3) That mice treated therapeutically with the PPARγ agonist, PGZ, are protected from lethal PR8 infection, and conversely, that the PPARγ^cKO^ mice are more susceptible, indicates that PPARγ activation is critical for M2 macrophage-mediated resistance to PR8 infection. (4) Our finding that PR8 infection resulted in inhibition of *Pparg* mRNA in mice, is consistent with those of Gopal et al. (42) and Huang et al. (25); however, we further observed that therapeutic PGZ administration to PR8-infected mice prevented the observed decrease in *Pparg* mRNA and paralleled the decrease in proinflammatory gene expression. In contrast to the findings of Huang et al. (25), however, we observed a significant increase in expression of the M2 gene, *Mrc1*, with a trend toward increased expression of *Arg1* mRNA, suggesting that activation of PPARγ by PGZ facilitates M2 gene expression with a concomitant inhibition of proinflammatory M1 gene expression. Finally, (5) our data in cotton rats infected with human pH1N1 shows that our observations are not restricted to murine models of influenza infection, and confirms that human influenza infection reduced *Pparg* mRNA expression, while PGZ treatment decreased proinflammatory mediators, resulting in greatly mitigated lung histopathology.

PPARγ/RXR heterodimers are required for the occupancy of canonical M2 promoters for polarization and regulation of the transcription of these genes (rev. in 45). However, the relative contribution of each ligand for the heterodimer in their transcriptional regulation has been studied and some results conflict with ours. Our data using peritoneal macrophages and the MH-S human alveolar macrophage cell line are in partial agreement to those of Daniel et al. (17), since activation of *Arg1* and *Mrc1* were PPARγ ligand-independent when a high dose of IL-4 was used for treatment of the cells (>5 ng/ml). However, at lower concentrations of IL-4 (1 and 5 ng/ml), RGZ and/or LG100754 enhanced *Arg1* and *Mrc1* gene expression, suggesting that differentiation into M2a macrophages could benefit from liganded PPARγ/RXR heterodimers (*i.e.*, they are not completely PPARγ/RXR ligand-independent genes). Furthermore, in our experiments using low IL-4 concentrations, the RXR ligand, LG100754 (in the absence or presence of RGZ), allowed the highest activation of *Arg1* and *Mrc1*. For our work, we utilized LG100754 as the ligand for RXR since it functions as an agonist for PPAR:RXR heterodimer-activated genes, but as an antagonist for RXR:RXR homodimer-activated genes (49). Using another RXR agonist (LG100268), Daniel et al. (17) did not confirm this ligand-dependent property of RXR in the heterodimer in M2 canonical genes. We cannot rule out the possibility that LG100754 liganded another receptor that heterodimerizes with RXR (*e.g*., RAR/RXR (50)) and/or that LG100754 and LG100268 have different agonist activity on the PPARγ contributing to the differing observations (*e.g.*, these two ligands may prompt PPARγ/RXR heterodimers to bind to different consensus sequences in the promoter regions of *Arg1* and *Mrc1*, and/or recruit different co-activators/co-repressors with different affinities for their activation domains). LG100754 was chosen because it is an RXR homodimer antagonist (50), does not activate key farnesoid X receptor and liver X receptor target genes (49), and it enhances the potency PPAR ligands (51, 52). It shows high affinity for RXRs (<15 nM) and binds RARs (retinoic acid receptors) with low affinity (>1,000 nM) (52). Still, some agonist activity of LG100754 was described for the RAR/RXR heterodimer and was explained by its binding to RXR to allosterically activate the heterodimer (53) or by a mild binding to RAR in ligand-dependent trypsin sensitivity assays and Gal-RAR chimeric reporter assays (50). Despite this potential overlap in specificities, no role of the RAR-RXR heterodimer was described for M2 polarization process. Pretreatment with low concentration of IL-4 for 24 hr, followed by incubation with RGZ and/or LG for additional 24 or 48 hr, resulted in ligand- and time-dependent increase in activation, suggesting that ligands were acting on heterodimers already positioned on the promoters of these genes (17). Since the study by Daniel et al. was carried out at 20 ng/ml IL-4, it is also possible that this high concentration of IL-4 (>5 ng/ml) elicits production of natural ligands for the heterodimer, skewing the effect of exogenous ligands. This observation is consistent with a reduced effect of the PPARγ and RXR ligands at higher concentrations of IL-4 as seen in our study (**Supplemental Figure 1**). Forman (51) studied the effects of LG100754 *in vitro* and reported that binding of this ligand to RXR increased the binding of RGZ and the natural ligand, 15-deoxy-D (12, 14)-prostaglandin J2 (54), suggesting that binding of this RXR ligand enhances the affinity of PPARγ for its ligands. It remains to be determined if this potential mechanism plays a role in our findings.

Increased influenza-induced lung pathogenesis is associated with sustained inflammatory and interferon responses, early influx of inflammatory cells including inflammatory macrophages, and an absence of induction of lipid metabolism (55). PPARγ, as well as other transcription factors involved in lipid metabolism (*e.g.*, hepatocyte nuclear factors), are reduced after influenza infection in mice (56) and correlate with the early production of type I IFN (25, 42). This is also in agreement with the low production of IL-4 during early influenza infection, and thus, a lack of STAT6-dependent induction of *Pparg* gene. Our data extend these results to influenza infection in other susceptible species (cotton rats) and show that treatment with the PPARγ agonist PGZ, reverses influenza-dependent inhibition of PPARγ expression (**Figures 2C, E**). Recently, in a model of cigarette smoke exposure in rats in which the M1 response is also exacerbated, both PPARγ and RXR expression were reduced in lung macrophages (56). Importantly, treatment with RGZ decreased the M1/M2 ratio of macrophages in the lung, reversing the repression of PPARγ/RXRα expression. Furthermore, the anti-inflammatory effect of RGZ was enhanced by the presence of the ligand for RXR (56). Although the mechanism by which RGZ or PGZ de-repress influenza-induced *Pparg* mRNA expression is not understood, previous studies have suggested that PPAR agonists also repress the activation of Type I (*Ifnb*) (57, 58) and Type II (*Ifng*) interferon genes (57). However, in our models of infection, we were unable to detect changes in the expression of interferon genes or early interferon-stimulated genes such as *Mx2* after PGZ treatment of influenza-infected animals (data not shown). In fact, contrary to other reports, influenza viral load was not affected as indicated by the expression of influenza M gene in control and PGZ-treated cotton rats (**Figure 3B**).

Among the unexpected findings in our report was the observation that neither *Chil3* nor *Retnla* were responsive to PPARγ or RXR ligands in IL-4-treated macrophages (**Figures 4E–H** and **5E–H**). However, Roulliard et al. (59) published a 755 gene data set entitled “CHEA Transcription Factor Binding Site Profiles” for PPARγ transcription factor binding evidence in thioglycollate-elicited macrophages. Both *Arg1* and *Mrc1*, but neither *Chil3* nor *Retnla*, were identified in this *in silico* search. Daniel et al. (18) reported that IL-4-induced, STAT6-dependent induction of *Arg1*, *Mrc1*, *Chil3*, and *Retnla*; however*, Chil3* and *Retnla* induction was regulated by Early Growth Response Gene 2 (EGR2), while *Arg1* and *Mrc1* gene expression were EGR2-independent. In studies carried out by Polumuri et al. (60), IL-4-induced *Arg1* and *Mrc1* gene expression were augmented by cAMP agonists, yet *Chil3* and *Retnla* were not (unpublished observation). Thus, while certain IL-4-inducible M2a genes are PPARγ-dependent, these findings suggest that others are not. The importance of this differential dependence on PPARγ for IL-4-inducible gene expression in the final phenotype of M2a macrophages will be a matter for future study.

TSPO has been shown to be strongly upregulated by M1 stimuli and down-regulated by M2 stimuli. Zhou et al. (48) further showed that M2 polarization of microglia by IL-4 resulted in decreased TSPO, while *PPARg* mRNA and PPARγ protein levels were increased. Agonist-induced activation of TSPO or TSPO overexpression was sufficient to repress the IL-4-induced expression of PPARγ (48). Thus, the observation that IL-4-induced *Arg1* and *Mrc1* mRNA were down-regulated in the presence of a TSPO agonist (**Figure 7**) further supports the notion that PPARγ regulates expression of these two genes; however, the unexpected finding that *Retnla* mRNA was also down-regulated suggests that TSPO may regulate other signaling pathways that are independent of PPARγ. In this regard, early work by Odegard et al. (22) provided evidence that in addition to PPARγ, the PPARδ isoform is also required for induction of M2 macrophage markers, although this may be dependent, in part, on the specific macrophage population being studied. Nonetheless, it is tempting to speculate that TSPO activation modulates M2 gene expression by altering the relative expression of PPARγ *vs*. PPARδ.

An early study using RGZ and PGZ prophylactically showed that treatment with both compounds protected mice from lethal PR8 challenge (61). In another study, treatment of mice lethally challenged with influenza with the prostanoid 15-deoxy-D (12, 14)-prostaglandin-J2, an endogenous PPARγ agonist (54), starting one day after infection was protective, but was not efficacious when treatment was initiated the same day of infection (62). Although these results are in agreement with the protective role of PPARγ against influenza, they also suggest variability in the different PPARγ ligands in their efficacy and suggest that the kinetics of activation of the IL-4/STAT-6 signaling pathway by influenza infection could be a critical factor.

## Data availability statement

The original contributions presented in the study are included in the article/**Supplementary Materials**. Further inquiries can be directed to the corresponding author.

## Ethics statement

The animal study was reviewed and approved by IACUC, University of Maryland Baltimore and Sigmovir Biosystems, Inc.

## Author contributions

SV, AK, and JB designed the overall study, with AG, JJ, KS, and MB contributing to design and execution of specific experiments. All authors contributed to the writing of this manuscript and approved the submitted version.

## Funding

This work was supported by NIH AI123371 (SV), AI143845 (AK/SV), AI163543 (SV/JB) and AI159507 (KS).

## Conflict of interest

Authors JJ, MB, and JB are employed by Sigmovir Biosystems, Inc.

The remaining authors declare that the research was conducted in the absence of any commercial or financial relationships that could be construed as a potential conflict of interest.

## Publisher’s note

All claims expressed in this article are solely those of the authors and do not necessarily represent those of their affiliated organizations, or those of the publisher, the editors and the reviewers. Any product that may be evaluated in this article, or claim that may be made by its manufacturer, is not guaranteed or endorsed by the publisher.

## References

[1] Mills EL O'Neill LA. Reprogramming mitochondrial metabolism in macrophages as an anti-inflammatory signal. Eur J Immunol (2016) 46(1):13–21. doi: 10.1002/eji.201445427 26643360

[2] Prantner D Nallar S Vogel SN. The role of RAGE in host pathology and crosstalk between RAGE and TLR4 in innate immune signal transduction pathways. FASEB J (2020) 34(12):15659–74. doi: 10.1096/fj.202002136R PMC8121140 33131091

[3] Mosser DM Edwards JP. Exploring the full spectrum of macrophage activation. Nat Rev Immunol (2008) 8(12):958–69. doi: 10.1038/nri2448 PMC2724991 19029990

[4] Gordon S. Alternative activation of macrophages. Nat Rev Immunol (2003) 3(1):23–35. doi: 10.1038/nri978 12511873

[5] Anthony RM Rutitzky LI Urban JF Jr. Stadecker MJ Gause WC. Protective immune mechanisms in helminth infection. Nat Rev Immunol (2007) 7(12):975–87. doi: 10.1038/nri2199 PMC2258092 18007680

[6] Shirey KA Pletneva LM Puche AC Keegan AD Prince GA Blanco JCG. Control of RSV-induced lung injury by alternatively activated macrophages is IL-4R alpha-, TLR4-, and IFN-beta-dependent. Mucosal Immunol (2010) 3(3):291–300. doi: 10.1038/mi.2010.6 20404812 PMC2875872

[7] Mills CD Ley K. M1 and M2 macrophages: the chicken and the egg of immunity. J Innate Immun (2014) 6(6):716–26. doi: 10.1159/000364945 PMC4429858 25138714

[8] Davies LC Rice CM Palmieri EM Taylor PR Kuhns DB McVicar DW. Peritoneal tissue-resident macrophages are metabolically poised to engage microbes using tissue-niche fuels. Nat Commun (2017) 8(1):2074. doi: 10.1038/s41467-017-02092-0 29234000 PMC5727035

[9] Davies LC Rice CM McVicar DW Weiss JM. Diversity and environmental adaptation of phagocytic cell metabolism. J Leukoc Biol (2019) 105(1):37–48. doi: 10.1002/JLB.4RI0518-195R 30247792 PMC6334519

[10] Lachmandas E Boutens L Ratter JM Hijmans A Hooiveld GJ Joosten LA. Microbial stimulation of different toll-like receptor signalling pathways induces diverse metabolic programmes in human monocytes. Nat Microbiol (2016) 2:16246. doi: 10.1038/nmicrobiol.2016.246 27991883

[11] Richard K Piepenbrink KH Shirey KA Gopalakrishnan A Nallar S Prantner DJ. A mouse model of human TLR4 D299G/T399I SNPs reveals mechanisms of altered LPS and pathogen responses. J Exp Med (2021) 218(2):e20200675. doi: 10.1084/jem.20200675 33216117 PMC7685774

[12] Murray PJ Allen JE Biswas SK Fisher EA Geilroy DW Goerdt S. Macrophage activation and polarization: nomenclature and experimental guidelines. Immunity (2014) 41(1):14–20. doi: 10.1016/j.immuni.2014.06.008 25035950 PMC4123412

[13] Pisu D Huang L Grenier JK Russell DG. Dual RNA-seq of mtb-infected macrophages *in vivo* reveals ontologically distinct host-pathogen interactions. Cell Rep (2020) 30(2):335–50.e4. doi: 10.1016/j.celrep.2019.12.033 31940480 PMC7032562

[14] Russell DG Huang L VanderVen BC. Immunometabolism at the interface between macrophages and pathogens. Nat Rev Immunol (2019) 19(5):291–304. doi: 10.1038/s41577-019-0124-9 30679807 PMC7032560

[15] Svedberg FR Brown SL Krauss MZ Campbell L Sharpe C Clausen M. The lung environment controls alveolar macrophage metabolism and responsiveness in type 2 inflammation. Nat Immunol (2019) 20(5):571–80. doi: 10.1038/s41590-019-0352-y PMC8381729 30936493

[16] Zhou B Magana L Hong Z Huang LS Chakraborty S Tsukasaki Y. The angiocrine Rspondin3 instructs interstitial macrophage transition *via* metabolic-epigenetic reprogramming and resolves inflammatory injury. Nat Immunol (2020) 21(11):1430–43. doi: 10.1038/s41590-020-0764-8 PMC7815054 32839607

[17] Daniel B Nagy G Czimmerer Z Horvath A Hammers DW Cuaranta-Monroy I. The nuclear receptor PPARgamma controls progressive macrophage polarization as a ligand-insensitive epigenomic ratchet of transcriptional memory. Immunity (2018) 49(4):615–26.e6. doi: 10.1016/j.immuni.2018.09.005 30332629 PMC6197058

[18] Daniel B Czimmerer Z Halasz L Boto P Kolostyak Z Poliska S. The transcription factor EGR2 is the molecular linchpin connecting STAT6 activation to the late, stable epigenomic program of alternative macrophage polarization. Genes Dev (2020) 34:1474–92. doi: 10.1101/gad.343038.120 PMC7608752 33060136

[19] Xavier MN Winter MG Spees AM den Hartigh AB Nguyen K Roux CM. PPARgamma-mediated increase in glucose availability sustains chronic brucella abortus infection in alternatively activated macrophages. Cell Host Microbe (2013) 14(2):159–70. doi: 10.1016/j.chom.2013.07.009 PMC3777723 23954155

[20] Malur A McCoy AJ Arce S Barna BP Kavuru MS Malur AG. Deletion of PPAR gamma in alveolar macrophages is associated with a Th-1 pulmonary inflammatory response. J Immunol (2009) 182(9):5816–22. doi: 10.4049/jimmunol.0803504 19380830

[21] Welch JS Escoubet-Lozach L Sykes DB Liddiard K Greaves DR Glass CK. TH2 cytokines and allergic challenge induce Ym1 expression in macrophages by a STAT6-dependent mechanism. J Biol Chem (2002) 277(45):42821–9. doi: 10.1074/jbc.M205873200 12215441

[22] Odegaard JI Ricardo-Gonzalez RR Goforth MH Morel CR Subramanian V Mukundan L. Macrophage-specific PPARgamma controls alternative activation and improves insulin resistance. Nature (2007) 447(7148):1116–20. doi: 10.1038/nature05894 PMC2587297 17515919

[23] Shirey KA Lai W Pletneva LM Finkelman FD Feola DJ Blanco JC. Agents that increase AAM differentiation blunt RSV-mediated lung pathology. J Leukoc Biol (2014) 96(6):951–5. doi: 10.1189/jlb.4HI0414-226R PMC4226793 25009233

[24] Schneider C Nobs SP Heer AK Kurrer M Klinke G van Rooijen N. Alveolar macrophages are essential for protection from respiratory failure and associated morbidity following influenza virus infection. PloS Pathog (2014) 10(4):e1004053. doi: 10.1371/journal.ppat.1004053 24699679 PMC3974877

[25] Huang S Zhu B Cheon IS Goplen NP Jiang L Zhang R. PPARγ in macrophages limits pulmonary inflammation and promotes host recovery following respiratory viral infection. J Virol (2019) 93(9):e00030–19. doi: 10.1128/JVI.00030-19 PMC6475778 30787149

[26] Teijaro JR Njau MN Verhoeven D Chandran S Nadler SG Hasday J. Costimulation modulation uncouples protection from immunopathology in memory T cell responses to influenza virus. J Immunol (2009) 182(11):6834–43. doi: 10.4049/jimmunol.0803860 19454679

[27] Shirey KA Lai W Scott AJ Lipsky M Mistry P Pletneva LM. The TLR4 antagonist eritoran protects mice from lethal influenza infection. Nature (2013) 497(7450):498–502. doi: 10.1038/nature12118 23636320 PMC3725830

[28] Shirey KA Lai W Patel MC Pletneva LM Pang C Kurt-Jones E. Novel strategies for targeting innate immune responses to influenza. Mucosal Immunol (2016) 9(5):1173–82. doi: 10.1038/mi.2015.141 PMC5125448 26813341

[29] Perkins DJ Richard K Hansen AM Lai W Nallar S Koller B. Autocrine-paracrine prostaglandin E2 signaling restricts TLR4 internalization and TRIF signaling. Nat Immunol (2018) 19:1309–18. doi: 10.1038/s41590-018-0243-7 PMC6240378 30397349

[30] Cole LE Elkins KL Michalek SM Qureshi N Eaton LJ Rallabhandi P. Immunologic consequences of francisella tularensis live vaccine strain infection: role of the innate immune response in infection and immunity. J Immunol (2006) 176:6885–91. doi: 10.4049/jimmunol.176.11.6888 16709849

[31] Shirey KA Cole LE Keegan AD Vogel SN. Francisella tularensis live vaccine strain induces macrophage alternative activation as a survival mechanism. J Immunol (2008) 181:4159–67. doi: 10.4049/jimmunol.181.6.4159 PMC2637804 18768873

[32] Blanco JCG Richardson JY Darnell MER Rowzee A Pletneva L Porter DD. Cytokine and chemokine gene expression after primary and secondary respiratory syncytial virus infection in cotton rats. J Infect Dis (2002) 185(12):1780–5. doi: 10.1086/340823 12085325

[33] Livak KJ Schmittgen TD. Analysis of relative gene expression data using real-time quantitative PCR and the 2(-delta delta C(T)) method. Methods (2001) 25(4):402–8. doi: 10.1006/meth.2001.1262 11846609

[34] Chen WH Toapanta FR Shirey KA Zhang L Giannelou A Page C. Potential role for alternatively activated macrophages in the secondary bacterial infection during recovery from influenza. Immunol Lett (2012) 141(2):227–34. doi: 10.1016/j.imlet.2011.10.009 PMC3243824 22037624

[35] Kelly-Welch AE Hanson EM Boothby MR Keegan AD. Interleukin-4 and interleukin-13 signaling connections maps. Science (2003) 300(5625):1527–8. doi: 10.1126/science.1085458 12791978

[36] Stein M Keshav S Harris N Gordon S. Interleukin 4 potently enhances murine macrophage mannose receptor activity: a marker of alternative immunologic macrophage activation. J Exp Med (1992) 176(1):287–92. doi: 10.1084/jem.176.1.287 PMC2119288 1613462

[37] Liu T Jin H Ullenbruch M Hu B Hashimoto N Moore B. Regulation of found in inflammatory zone 1 expression in bleomycin-induced lung fibrosis: role of IL-4/IL-13 and mediation *via* STAT-6. J Immunol (2004) 173(5):3425–31. doi: 10.4049/jimmunol.173.5.3425 15322207

[38] Richardson JY Ottolini MG Pletneva L Boukhvalova MS Zhang S Vogel SN. Respiratory syncytial virus (RSV) infection induces cyclooxygenase 2 (COX-2) – a potential target for RSV therapy. J Immunol (2005) 174:4356–64. doi: 10.4049/jimmunol.174.7.4356 15778400

[39] Carey MA Bradbury JA Seubert JM Langenbach R Zeldin DC Germolec DR. Contrasting effects of cyclooxygenase-1 (COX-1) and COX-2 deficiency on the host response to influenza a viral infection. J Immunol (2005) 175:6878–84. doi: 10.4049/jimmunol.175.10.6878 16272346

[40] Carey MA Bradbury JA Rebolloso YD Graves JP Zeldin DC Germolec DR. Pharmacologic inhibition of COX-1 and COX-2 in influenza a viral infection in mice. PloS One (2010) 5(7):e11610. doi: 10.1371/journal.pone.0011610 20657653 PMC2904706

[41] Wagstaff AJ Goa KL. Rosiglitazone: A review of its use in the management of type 2 diabetes mellitus. Drugs (2002) 62(12):1805–37. doi: 10.2165/00003495-200262120-00007 12149047

[42] Gopal R Mendy A Marinelli MA Richwalls LJ Seger PJ Patel S. Peroxisome proliferator-activated receptor gamma (PPAR) suppresses inflammation and bacterial clearance during influenza-bacterial super-infection. Viruses (2019) 11(6):505, 1–8. doi: 10.3390/v11060505 PMC6630660 31159430

[43] Ottolini MG Blanco JGC Eichelberger MC Porter DD Pletneva L Richardson JY. The cotton rat provides a useful small animal model for the study of influenza virus pathogenesis. J Gen Virol (2005) 86:2823–30. doi: 10.1099/vir.0.81145-0 16186238

[44] Blanco JC Pletneva LM Wan H Araya Y Angel M Oue RO. Receptor characterization and susceptibility of cotton rats to avian and 2009 pandemic influenza virus strains. J Virol (2013) 87(4):2036–45. doi: 10.1128/JVI.00638-12 PMC3571489 23192875

[45] Toobian D Ghosh P Katkar GD. Parsing the role of PPARs in macrophage processes. Front Immunol (2021) 12:783780. doi: 10.3389/fimmu.2021.783780 35003101 PMC8727354

[46] Kurokowa R Yu VC Naar A Kyakumoto S Han Z Silverman S. Differential orientations of the DNA-binding domain and carboxy-terminal dimerization interface regulate binding site selection by nuclear receptor heterodimers. Genes Dev (1993) 7(7B):1423–35. doi: 10.1101/gad.7.7b.1423 8392479

[47] Yao R Pan R Shang C Li X Cheng J Xu J. Translocator protein 18 kDa (TSPO) deficiency inhibits microglial activation and impairs mitochondrial function. Front Pharmacol (2020) 11:986. doi: 10.3389/fphar.2020.00986 32695005 PMC7339871

[48] Zhou D Ji L Chen Y. TSPO modulates IL-4-induced microglia/macrophage M2 polarization *via* PPAR-γ pathway. J Mol Neurosci (2020) 70:542–9. doi: 10.1007/s12031-019-01454-1 31879837

[49] Cesario RM Klausing K Razzaghi H Crombie D Rungta D Heyman RA. The rexinoid LG100754 is a novel RXR:PPARγ agonist and decreases glucose levels in vivo. Mol Endocrinol (2001) 15(8):1360–9. doi: 10.1210/mend.15.8.0677 11463859

[50] Le Maire A Teyssier C Balaguer P Bourguet W Germain P. Regulation of RXR-RAR heterodimers by RXR- and RAR-specific ligands and their combinations. Cells (2019) 8:1392. doi: 10.3390/cells8111392 PMC6912802 31694317

[51] Forman BM. The antidiabetic agent LG100754 sensitizes cells to low concentrations of peroxisome proliferator-activated receptor gamma ligands. J Biol Chem (2002) 277(15):12503–6. doi: 10.1074/jbc.C200004200 11877384

[52] Lala DS Mukherjee R Schulman IG Koch SS Dardashti LJ Nadzan AM. Activation of specific RXR heterodimers by an antagonist of RXR homodimers. Nature (1996) 383(6599):450–3. doi: 10.1038/383450a0 8837780

[53] Sato Y Ramalanjaona N Huet T Potier N Osz J Antony P. The “Phantom effect” of the rexinoid LG100754: Structural and functional insights. PloS One (2010) 5:e15119. doi: 10.1371/journal.pone.0015119 21152046 PMC2994906

[54] Kliewer SA Lenhard JM Wison TM Patel I Morris DC Lehmann JM. A prostaglandin J2 metabolite binds peroxisome proliferator-activated receptor gamma and promotes adipocyte differentiation. Cell (1995) 83(5):813–9. doi: 10.1016/0092-8674(95)90194-9 8521498

[55] Josset L Belser JA Pantin-Jackwood MJ Chang JH Chang ST Belisle SE. Implication of inflammatory macrophages, nuclear receptors, and interferon regulatory factors in increased virulence of pandemic 2009 H1N1 influenza a virus after host adaptation. J Virol (2012) 86(13):7192–206. doi: 10.1128/JVI.00563-12 PMC3416346 22532695

[56] Feng H Yin Y Zheng R Kang J. Rosiglitazone ameliorated airway inflammation induced by cigarette smoke *via* inhibiting the M1 macrophage polarization by activating PPARγ and RXRα. Int Immunopharmacol (2021) 97:107809. doi: 10.1016/j.intimp.2021.107809 34182323

[57] Weber K Sauer M He L Tycksen E Kalugotla G Razani B. PPARγ deficiency suppresses the release of IL-1β and IL-1α in macrophages *via* a type 1 IFN–dependent mechanism. J Immunol (2018) 201:2054–69. doi: 10.4049/jimmunol.1800224 PMC6147147 30143592

[58] Zhao W Wang L Zhang M Wang P Zhang L Yuan C. Peroxisome proliferator-activated receptor gamma negatively regulates IFN-beta production in toll-like receptor (TLR) 3- and TLR4-stimulated macrophages by preventing interferon regulatory factor 3 binding to the IFN-beta promoter. J Biol Chem (2011) 286:5519–28. doi: 10.1074/jbc.M110.149823 PMC3037665 21148557

[59] Roulliard AD Gundersen GW Fernandez NF Wang Z Monteiro CD McDermott MG. The harmonizome: a collection of processed datasets gathered to serve and mine knowledge about genes and proteins. Database (Oxford) (2016), baw100. doi: 10.1093/database/baw100 27374120 PMC4930834

[60] Polumuri S Perkins DJ Vogel SN. cAMP levels regulate macrophage alternative activation marker expression. Innate Immun (2021) 27(2):133–42. doi: 10.1177/1753425920975082 PMC7882807 33241977

[61] Moseley CE Webster RG Aldridge JR. Peroxisome proliferator activated receptor and AMP-activated protein kinase agonists protect against lethal influenza virus challenge in mice. Influenza Other Respir Viruses (2010) 4:307–11. doi: 10.1111/j.1750-2659.2010.00155.x PMC3584640 20716159

[62] Cloutier A Marois I Cloutier D Verreault C Cantin AM Richter MV. The prostanoid 15-Deoxy-12,14-Prostaglandin-J2 reduces lung inflammation and protects mice against lethal influenza infection. J Infect Dis (2012) 205:621–30. doi: 10.1093/infdis/jir804 22219346

