# Supplementary material for: Protection against influenza-induced Acute Lung Injury (ALI) by enhanced induction of M2a macrophages: possible role of PPARγ/RXR ligands in IL-4-induced M2a macrophage differentiation
Source: Front Immunol. 2022 Aug 16;13:968336. doi: 10.3389/fimmu.2022.968336 (PMC9424652; doi:10.3389/fimmu.2022.968336)
Supplement: Supplementary file 1 [file DataSheet_1.pdf]

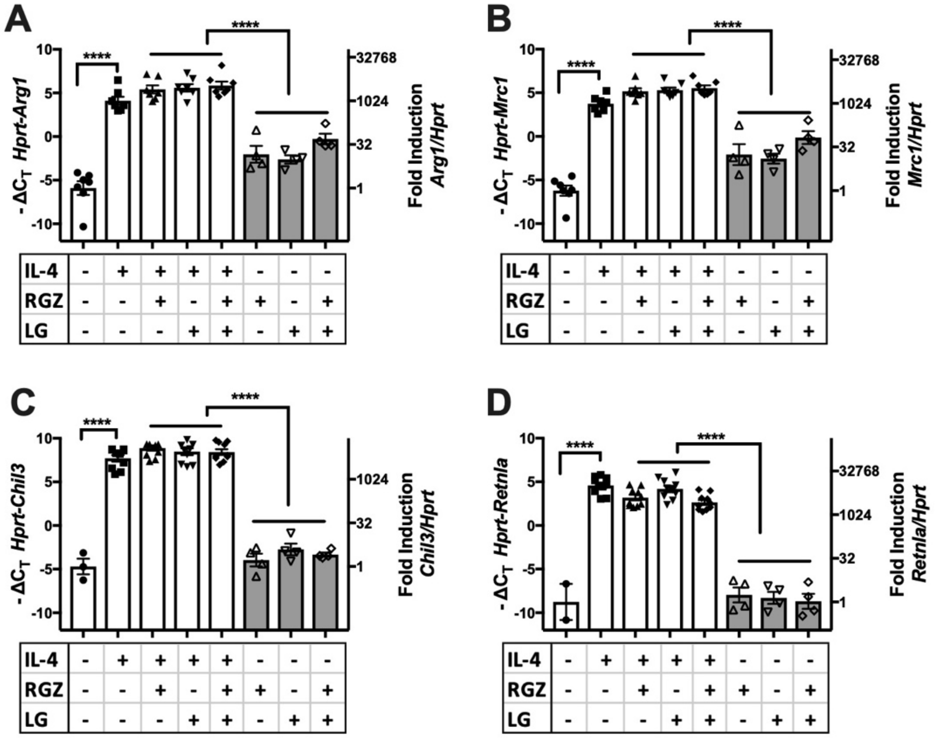

Supplementary Figure 1. Effect of IL-4, PPARγ and RXR agonists on M2 macrophage gene expression. Thioglycollate-elicited peritoneal macrophages were harvested from 6-8 week old WT C57BL/6J mice and stimulated with IL-4 at 20 ng/ml in the absence or presence of 1 μM of rosiglitazone (RGZ) (PPARγ ligand) and/or LG100754 (LG) (RXR ligand) for 48 hr, and RNA was processed as described in Methods. Gene expression of *Arg1* (**A**), *Mrc1* (**B**), *Chil3* (**C**), and *Retnla* (**D**) was quantified by qRT-PCR. Data were pooled from 7 independent experiments and is presented as $-\Delta C_T$ (left y-axis) and fold induction ($2^{-\Delta\Delta C_T}$ value; right y-axis), mean $\pm$ SEM. Statistical analysis (one-way ANOVA with Tukey's multiple comparison post-hoc test) was performed on the $-\Delta C_T$ values. ****$p < 0.0001$

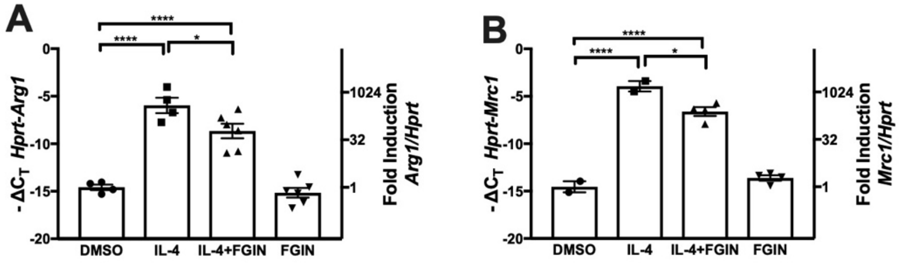

Supplementary Figure 2. Effect of TSPO agonist on IL-4-induced M2 macrophage gene expression

Murine alveolar macrophage cell line, MH-S was stimulated with IL-4 at 5 ng/ml in the absence or presence of 10 μM of FGIN-1-27 (TSPO agonist) for 48 hr, and RNA was processed. Gene expression of *Arg1* (**A**) and *Mrc1* (**B**) were quantified by qRT-PCR. Data from 2 independent experiments is presented as $-\Delta C_T$ (left y-axis) and fold induction ($2^{-\Delta\Delta C_T}$ value; right y-axis), mean $\pm$ SEM. Statistical analysis (one-way ANOVA with Tukey's multiple comparison post-hoc test) was performed on the $-\Delta C_T$ values. ****p <0.0001 and *p<0.05.
